# Supplementary material for: Generation of nanobodies acting as silent and positive allosteric modulators of the α7 nicotinic acetylcholine receptor
Source: Cell Mol Life Sci. 2023 May 25;80(6):164. doi: 10.1007/s00018-023-04779-8 (PMC10213069; doi:10.1007/s00018-023-04779-8)
Supplement: Supplementary file 1 — Supplementary file1 (DOCX 170 KB) [file 18_2023_4779_MOESM1_ESM.docx]

**Generation of nanobodies acting as silent and positive allosteric modulators of the α7 nicotinic acetylcholine receptor**

*Cellular and Molecular Life Sciences*

Qimeng Li^1,2,3,4^, Ákos Nemecz^1*^, Gabriel Aymé^2*^, Gabrielle Dejean de la Bâtie^1^, Marie S Prevost^1^, Stéphanie Pons^5^, Nathalie Barilone^1^, Rayen Baachaoui^1,2^, Uwe Maskos^5^, Pierre Lafaye^2^, Pierre-Jean Corringer^1*^

1 Institut Pasteur, Université Paris Cité, CNRS UMR 3571, Channel-Receptors Unit, Paris, France

2 Institut Pasteur, Université Paris Cité, CNRS UMR 3528, Antibody Engineering platform, Paris, France

3 Lanzhou Institute of Biological Product Co., Lanzhou, China

4 Sorbonne Université, Collège doctoral, Paris, France

5 Institut Pasteur, Université Paris Cité, CNRS UMR 3571, Integrative Neurobiology of Cholinergic Systems Unit, Paris, France

Corresponding authors*

[akos@umk.pl](mailto:akos@umk.pl)

[gabriel.ayme@pasteur.fr](mailto:Pierre.Lafaye@pasteur.fr)

[pjcorrin@pasteur.fr](mailto:pjcorrin@pasteur.fr)


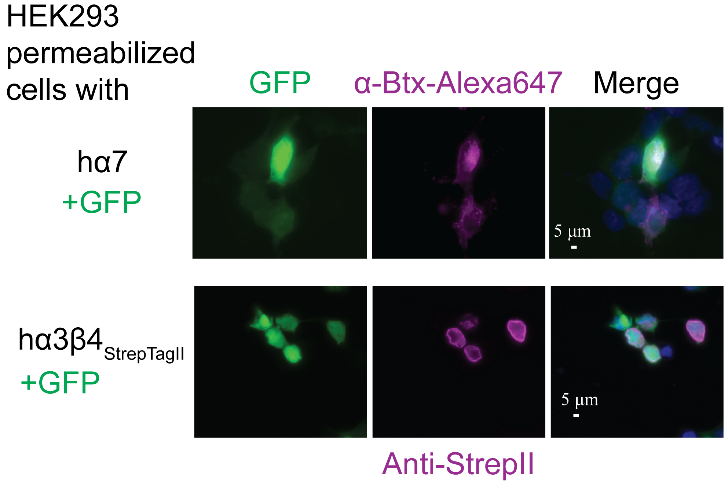


**Supplementary Fig 1** Immunofluorescence expression controls

Representative (of n=4) images of permeabilized HEK 293 cells expressing hα7- (top) and hα3hβ4_StrepII_- (bottom) nAChRs immunostained using conjugated α-Btx-Alexa Fluor™ 647 and an anti-StrepII tag detected by a conjugated anti-mouse IgG-Alexa Fluor™ 647 resp.. Dapi, shown in blue, stains the cells’ nucleus; Alex Fluor™ 647, a red wavelength, is colored as magenta. Cytoplasmic eGFP indicates efficiently transfected cells. Identical exposure times were used to visualize each channel on all conditions.
